# Moral equality and success of common-pool water governance in Namibia

**DOI:** 10.1007/s13280-016-0766-9

**Published:** 2016-02-16

**Authors:** Michael Schnegg, Michael Bollig, Theresa Linke

**Affiliations:** 1 Institute of Social and Cultural Anthropology, Universität Hamburg, 20146 Hamburg, Germany; 2 Institute of Social and Cultural Anthropology, Universität zu Köln, 50923 Köln, Germany

**Keywords:** Common-pool resource governance, Water, Success, Equality, Cost and benefit sharing, Namibia

## Abstract

In the course of decentralization, pastoral communities in Namibia have had to find new ways to share their most salient resource, water, and the costs involved in providing it. Using data from sixty communities, we examine (1) whether and to what extent different sharing rules emerge, (2) how variations can be explained, (3) how rules are perceived and influence success, and (4) what economic consequences they have. Our results reveal that either all members pay the same (numerical equality) or payment is according to usage (proportional equality). We find that although proportional equality provides more success, the rule can only pertain where the state maintains an active role. Simulations show that where it does not prevail, wealth inequality is likely to grow. These findings have political implications and suggest that, in the context of the widespread decentralization policies, the state should not withdraw if it aims to ensure the success of common-pool resource management and to fight poverty.

## Introduction

As the impact of humans on communal resources grows, there is an increasing need to better understand how resources can be governed sustainably. Elinor Ostrom has identified eight principles that explain failure and success in shared communal resource management. While the significance of some of the original variables (e.g., monitoring, sanctioning) has been explored to some extent during the last decades (Ostrom 1990; Agrawal 2001, 2003; Dietz et al. 2003; Anderies et al. 2004; Pagdee et al. 2006; Ostrom 2009; Cox et al. 2010; Poteete et al. 2010; Yang et al. 2013), comparably little is known about the efficacy of different cost- and benefit-sharing agreements (principle two in Ostrom’s list). At the same time, their significance gains weight. Over the last decades, nation states have increasingly withdrawn from local resource governance. Instead, legislation and post-Rio policies in the global South support community-based natural resource management (CBNRM) approaches, putting questions of best practices center stage (Brosius et al. 1998; Leach et al. 1999; Ribot 1999; Cleaver 2012; Hall et al. 2014; Cleaver and de Koning 2015).

Sharing agreements are based on notions of equality. Equality is a functional relationship between two variables, for example, time worked and payment received. According to Aristotle, there are two kinds of equality: *numerical* and *proportional* (Aristotle, *Nicomachean Ethics*, 1130b–1132b). With proportional equality, the payment received varies according to the time each individual spends actually working, whereas with numerical equality all those who work are paid the same amount. Logically, the latter is a special case of the former. The parable of the workers in the vineyard offers an illustration (Matthew 20:1–16). Here, the winegrower decided to pay all workers the same, independent of when they began working during the day. Thus, he established a relationship between the two variables at stake, ‘work’ and ‘pay’ which is not proportional but numerical in Aristotle’s sense. Not surprisingly, those who had been working since dawn complained.

In the case of communal resource governance, cost- and benefit-sharing institutions must resolve the same fundamental issue (Mahanty et al. 2009). With cost sharing, the appropriators have to agree whether they will each contribute the same, or whether each individual will pay according to the amount personally used. With benefit sharing, community members must likewise decide whether all should benefit equally, or whether those who invested more should also receive more.

The issue is central in Ostrom’s pioneering analysis (principle two), and she concluded that institutions are perceived to be fair if there is a ‘congruence between appropriation and provision rules’ (Ostrom 1990, p. 90), and that institutions perceived to be fair are more likely to be successful. The observation was confirmed in a number of case studies (Klooster 2000; Pomeroy et al. 2001; Trawick 2001). In their comprehensive review of Ostrom’s design principles, Cox et al. (2010) reformulated her second principle, stating that ‘The benefits obtained by users from a CPR [common-pool resource], as determined by appropriation rules, are proportional to the amount of inputs required in the form of labor, material, or money, as determined by provision rules.’ Based on their review of the literature published since 1990, the congruence is spelled out even more explicitly. From these theoretical and empirical findings, we expect that (1) an approach based on proportional cost/benefit sharing is more successful in the long run and (2) that the rule which is perceived to be fair is also more likely to prevail.

Currently, Namibia offers a unique opportunity to test these expectations in an investigation of the emergence, success, and consequences of sharing agreements on a large scale. In the course of decentralization, rural communities have had to find new ways to share their most salient resource, water, and the costs involved in providing it. In the arid environment of northwestern Namibia, pastoralism is the dominant livelihood strategy, and almost all households own cattle and small stock. Until some 50 years ago, most African pastoralists obtained water through natural springs, surface water, and hand-dug wells (McCabe 2004; Robinson 2009; Bollig 2013). Open water sources were usually managed with adjoining pastures (McCabe 2004). These conditions changed significantly in the middle of the twentieth century under the influence of the colonial state and its ‘modernization’ paradigm. Now, in many parts of Africa, boreholes are drilled and groundwater is withdrawn for household and livestock consumption. Extensive pastures previously only viable during or shortly after the rainy season when seasonal rivers and filled pans were abundant now became available year round. This ‘hydrological revolution’ allowed residents to sustain higher stocking numbers and altered mobility patterns significantly, often laying the basis for a more sedentary lifestyle (Picardi and Seifert 1976; Sobania 1988; Bollig 2013).

In northwestern Namibia, between 1960 and 1990 the number of boreholes increased almost by a factor of ten (Bollig 2013). Until independence in 1990, maintenance costs for rural water supply were born by the South West Africa administration under the jurisdiction of the colonial South African state. As long as the state covered the costs for establishing, running, and maintaining the infrastructure, little local coordination was required.

Starting in the mid 1990s, the implementation of CBNRM has led to a drastic reconfiguration of the organizational and institutional landscape (Barnes et al. 2002; Falk et al. 2009; Silva and Mosimane 2013; Bollig and Menestrey Schwieger 2014). A shift toward self-governance meant turning ownership of and responsibility for boreholes and rural water supplies over to user associations.^1^ As a result, hundreds of communities have had to devise rules for sharing the costs and benefits involved. The costs include diesel fuel to run engine pumps and paying for necessary repairs. As previous work has shown, this process has opened new paths to participation for rural communities and their inhabitants. At the same time, putting the economic responsibility in the hands of users creates an additional financial burden, which is hard to shoulder, especially for the poor (Falk et al. 2009). Even the Namibian Government admitted self-critically, that those costs can have negative effects and is considering subsidization strategies for poor farmers (Namibia 2000; Gildenhuys 2010). However, no further steps in this direction have been taken.

While previous scholarly and policy papers have pointed toward an increase in costs, it is not known to what extent and why different institutional regimes emerge and what distinct economic and social consequences different rules are likely to have. To address both questions, a larger sample of observations (communities), longitudinal data, or simulations is appropriate. Such data were not available prior to the study reported here. We explore for the first time (1) whether and to what extent different sharing rules emerge, (2) how possible variations can be explained, (3) how different rules are perceived and hence influence success in community water management, and (4) what economic consequences distinct rules are likely to have.

## Materials and methods

### Study area

The arid Kunene region is sparsely populated. Small communities dot the vast landscape and, on average, they contain 13.1 (SD 3.5) households with 13 (SD 9.3) household members each. In general, dependency on natural resources is high. Pastoralism is the dominant subsistence strategy and the pastoral livelihood is constrained by the environment, most notably the low and unpredictable precipitation (Bollig 2006; Schnegg et al. 2013). Annual rainfall varies around 200–300 mm and occurs in summer, between November and April (Schnegg and Bollig 2016). Under these ecological constraints, more than 25–30 ha of land is needed to sustain one head of cattle (Burke 2004). Water is provided through boreholes, often drilled more than 100 m deep. Today, the technology varies and in 80 percent out of the 60 communities we study the pumps are powered by diesel engines, whereas in 20 % solar panels drive electric motors. In the course of decentralization, the technological infrastructure of the boreholes was renewed and is comparably good across the communities we study.^2^ While the techncial running costs of solar panels are lower, they must be guarded against theft and repairs are more expensive. A head of cattle drinks about 27 l a day, whereas goats/sheep need only 2.2 l (Wilson 2007, p. 60f). For comparison, if the water is not piped to a house humans use about 20 l of water during the dry season (Linke 2015). With herds often exceeding 50 animals per household, largely cattle, the amount of water used for animals is thus significantly higher than human consumption.

Livestock possession is unequally distributed in Kunene and other parts of Namibia (Schnegg et al. 2013). We find in almost all communities at least one household that owns more than 100 cattle and we equally find one, and often more, owning less than 10 (mean LSU^3^ = 79.08, SD = 75.07, min = 2.33, max = 355.66). The Gini coefficient for livestock possessions per household, the most important economic asset, is 0.49. This coefficient falls in the range of what Falk et al. (2009) reported for other communities and is much higher than the income Gini of most European countries where it varies between 20 and 30 (UNDP 2014).

In terms of ethnicity, the study area is diverse. While most people in the northern-most region of our study area consider themselves as Ovahimba, the central region is inhabited principally by Ovaherero, and the southern communities by Damara/Nama. At the same time, most communities are ethnically mixed and we did not observe any effects between the main ethnic group in an area and the ways the water sharing process was experienced and managed.

In relatively small communities, people interact fact to face, and typically over 80 percent of the members of each community are related by kinship (Schnegg and Linke 2015). Within kinship networks, elder people typically occupy a special position. They possess most livestock, the central economic asset in pastoral communities. Livestock not only symbolize wealth and status, but also transfer directly into patron–client relationships, when, for example, cattle are lent to poorer relatives who herd them in exchange for the milk the cattle give.

In the course of decentralization, community-based management strategies are introduced in the communities by state officials. Rules and procedures follow a general script and clear recommendations about how to manage groundwater are given (Namibia 2006). Since the stimulus induced by decentralization was by and large the same throughout the research area, and technological, ecological, and socio-economic variables show little variation across the communities we study, the situation offered a unique opportunity to study the evolution of institutions from a comparative perspective.

### Ethnographic data

The data analyzed here were collected by a team of anthropologists between 2010 and 2012 (M. Bollig, M. Schnegg, Th. Kelbert, D. Menestrey, Th. Linke, K. Gradt) as part of a German Research Council (DFG)-funded research project LINGS (Local institutions in globalized societies). The two principle investigators, M. Schnegg and M. Bollig, have been conducting ethnographic fieldwork in the region since 1994 (M. Bollig) and 2002 (M. Schnegg) respectively, and are responsible for the overall design and comparative analysis of the data. In the first phase of the current fieldwork, three anthropologists (D. Menestrey, Th. Linke, K. Gradt) stayed for roughly 1 year between 2010 and 2011 in seven communities in the southern (Fransfontein), central (Otwani), and northern (Okangwati) parts of the research area to gain an in-depth understanding of processes entailed in negotiating and crafting new institutions through daily routines. During this time, all 80 households were interviewed about their social and economic livelihoods, including economic possessions and social networks.

### Comparative evidence from sixty communities

After an initial analysis of the ethnographic data collected during 2010 and 2011, we returned to the field in late 2012 to conduct the ‘upscaling’ research we had designed to study the distribution of some of the phenomena found in the community ethnographies. Since our study treats communities as cases, it is challenging to collect relatively large numbers of observations that permit meaningful comparisons. To facilitate this requirement, we designed an interview guide to elicit information at the community level. For geographical areas of approximately 250 km^2^ around Fransfontein, Otwani, and Okangwati, all communities were interviewed. We can thus treat the data as a complete sample for those three areas. We decided against a representative sample of the entire Kunene region due to its size, bad road infrastructure, logistical constraints, and the lack of a list of communities that could serve as a sampling frame. In addition, our approach allowed us to make use of the fact that fieldworkers were already known and trusted in the target areas.

The research protocol contained three sections. First, we elicited the rules of water management and the composition of community-based organizational structures for water governance in the community. Each interview took place in public and included both female and male informants, some of whom were active in the water point committee. The second and third sections dealt with the success of the water management and the conflicts communities had experienced or observed. Since those questions are more confidential, interviews were done in private, aiming at a sample of males and females of different age groups and from different economic positions. In total, we researched sixty communities. Since information remains incomplete for some of them, the number of communities included in the analysis is sometimes lower than that.

All group interviews were taped and two independent interview protocols were written by the main researcher and his/her assistant. Discrepancies were resolved thereafter. The data were entered in MySQL database, designed for the project. The coding was done by the principal investigators in collaboration with the researchers.

The measurement of success derives directly from the questions posed and is coded on an ordinal three-point scale. To measure the involvement of the state, we use two indicators. During the interview, we collected two types of information. First, the frequency and purpose of visits by state officials in the community during the last 2 years. If the state officials had visited the community at least once during the last year for consultancies and activities other than urgent repairs of broken infrastructure, we took this as an indication of more than average state involvement. Since CBNRM and the decentralization strategy are highly standardized, we did not observe any variations in the type of rules recommended. Generally, state officials try to push communities in certain directions above all concerning the payment schemes. Second, our ethnographic observations have shown that if employees of the ministry or commissioned NGOs lived in the communities at issue, the impact of the state was significantly stronger, because those people typically wanted their communities to be flagship cases for the state’s mission and ideology (see below). For the analysis, we coded the state involvement to be above average, if either of the two indicators was given.

## Results

### Cost- and benefit-sharing arrangements

The decentralization process in Namibia is carried out by extension officers from the regional authorities (Directorate of Rural Water Supply, DRWS) and/or NGOs contracted by the government (Falk et al. 2009; Bollig and Menestrey Schwieger 2014; Schnegg and Bollig 2016). To standardize the process, a ‘Handbook for Water Point Committees’ was developed and distributed to the NGO and state representatives administering the process on the local level (Namibia 2006). The handbook and related documents describe the process to be taken step by step in eleven sections and propose institutional solutions to the community. Sharing the costs of water is one of the most salient problems in water governance. Since most pumps operate with diesel, the price of water is largely determined by the amount and price of diesel required for pumping it. In line with the idea that water is an economic good, the handbooks spell out in session 5 ‘Managing WPA Finance’: *‘*‘Recommended is a rate per head of large or small stock, each member paying a certain rate per head of large or small [stock] accordingly, as to raise enough money to sustain the water point” (Namibia 2006, p. 8). We refer to this arrangement as the proportional rule.

During the process of implementation, emerging institutional arrangements are negotiated with representatives of the ministry or contracted NGOs. For doing so, the representatives visit the communities and call for meetings during which the many pertinent questions, e.g., access, sanctions for violations of the rules agreed, and—often most importantly—payment schemes are discussed. State officials explain in qualitative interviews that in recent years they especially focus on payment schemes and recommend the proportional rule (Linke 2015). Since discussions about the payment scheme are typically conflictive, the process often requires a number of meetings that stretch over months. During the meetings, state representatives take an active role. They go through the sections of the above-mentioned handbook and sensitize the communities to the issues they have to resolve. Often, the moderator uses flip charts to summarize his or her input and that of the communities. At the end, a consensus is fixed in two documents: the ‘constitution’ and the ‘management plan’ containing information about the payment scheme.

In the communities we studied, two types of rules were applied. Among the fifty-six water management groups for which we have information, 25 (44.6 %) agreed that individuals paid fees according to the number of livestock they owned (e.g., 2 N$ per head of cattle and 1 N$ per goat/sheep per month). Thus, the more water one uses, the more they pay. This fits the notion of proportional equality. In addition, seven communities (12.5 %) used an attenuated form in which the rich paid more, but not exactly in proportion to the number of their livestock.^4^ However, in 24 communities (42.9 %) we found an institutional regime in which all households paid the same (e.g., 100 N$ per household per month), and which was therefore based on the principle of numerical equality.^5^ Thus, only about half of the observations confirm the existing literature that proportional equality is likely to emerge (Ostrom 1990, p. 90; Klooster 2000; Pomeroy et al. 2001; Trawick 2001; Cox et al. 2010).

Before we offer an explanation why, we want to rule out two common alternative hypotheses: technology and a “majority cost/benefit vote” (Ostrom 1990; Anderies et al. 2004). Whereas Anderies et al. (2004) have proposed that technology plays a central role for institutional design, we find a very low and non-significant relationship between the technological infrastructure (solar/diesel) and the payment regime (phi = 0.155, P = 0.244). Alternatively, Ostrom has argued that institutions tend to develop in ways that serve the majority (Ostrom 1990, p. 193). In our case, that would mean that households opt for the solution under which they pay less (given their number of animals) and that the community ends up with the solution that serves most households best. As a test of this hypothesis reveals, in most communities (88.9 %), the majority of households would profit financially from a proportional rule. Thus, the hypothesis of a rational majority vote can hardly explain why in a great many communities numerical equality is reality.

The account we provide instead is formulated on the basis of our ethnographic work and includes four factors. Of those, three apply in all communities and favor a numerical rule. Only the last factor, the involvement of the state, makes a difference, as the statistical analysis reveals.

### Micro-politics of water

#### Administration costs

To establish and maintain a proportional rule is more costly than a numerical rule. Most importantly, it requires counting animals. Counting livestock is difficult. Counting individual animals of small stock in large herds from different owners flowing toward a well is nearly impossible. In addition, there are cultural taboos against counting livestock, and people complain that counting livestock brings about bad luck (Bollig 2006). In general and across all communities, this favors numerical equality.

#### Wealth and bargaining power

Not surprising, wealthy herd owners oppose a proportional rule and opt for numerical equality (Menestrey Schwieger 2015). Often, this is justified by pointing out that the higher burden on the poor is balanced out through other exchanges, when, for example, Hermann explains to us: “Jorries who is having fifty cows is not only keeping them for the water, but he is also taking milk from his animals and gives this to you so you can prepare some porridge and eat it with the milk.” In contrast, most poor households argue like Justus who reasons: “There is now one house that we call Herbert Humbandi’s house. This house has a lot of cattle, maybe, over 300. [With the numerical rule] this household oppresses the others who have only small stock. I have only 8 cows. And then, I have to pump water, for that one who has 300 as well. For the whole month. This is very difficult. […]. So that’s why we say, if you have a lot of cattle, you have to pump more.”^6^

In the regional cultural context and across all three research sites, social status and bargaining power strongly correlate with economic status (Pauli 2011). Elderly men commonly occupy the positions at the top of this ladder. These positions are sustained by the material basis of the economy, cattle ownership, which is, as we have seen, unevenly distributed. Across all communities, there is ample ethnographic evidence that those who own more use their bargaining power to push for an institutional regime that is favorable to them (i.e., numerical equality). The nature of social ties is key to understanding why they often succeed (Schnegg and Linke 2015).

#### Multiplexity of ties and norms of sharing

The communities each consist of fewer than 20 households, and people interact in multiple ways and roles. Thus, sharing water can hardly be separated from the remaining social and economic aspects of life. People also interact as kin, as lenders and borrowers of animals, and as providers of other resources (e.g., car rides, advice, ritual services). Thus, water is only one resource in a larger sharing arrangement and a moral model exists that short-term imbalances in one domain will equal out across all types of transfers and time (Schnegg and Bollig 2016). Furthermore, sharing norms foster a common belief that every household of the community needs to contribute to sustain collective goods and show his cooperative commitment (Linke 2015; Schnegg 2015). Given this interconnectedness in multiple networks, it is practically impossible for the less wealthy to force those who are better off to pay more than the rest if the latter refuse (Schnegg and Linke 2015). They readily respond that even if they pay less for water, they provide many other goods for the community and especially the poor (e.g., transportation, milk). Again, this works in favor of numerical equality.

#### The Role of the state

Given the three dynamics just described, we would expect all communities to end up with a numerical equality rule. To understand why this is not the case, we have to take the state and its agents into account. As we have seen, and as it is expressed in the handbook and in qualitative interviews with extension officers, the state has an explicit preference for proportional equality. While the state does not provide any material incentives to apply proportional equality or penalize communities that opt for numerical rule, its representatives clearly state in public meetings that proportional equality is what the state favors and what they perceive to be just and fair. Unsurprisingly then, a first examination of those thirty-one cases that establish proportional equality reveals that in these cases Ministries and NGOs maintain strong involvement in the local water governance through regular visits and support.

An interview with Christa, who works for the Ministry of Agriculture, Water & Forestry and is responsible for a large number of water points, shows the state at work. When I confronted her with my observation that many communities switch to a numerical regime she responded: “It is not fair. But as soon as we turn our back the community big men come and tell the rest what to do.” In the course of the interview, she repeatedly states how hard it is for her, the official from the Ministry, to implement the rule in the community where she is farming herself. Asked where the proportional rule is actively working, she starts talking about the community Duurwater^7^ where an active young women is the chairperson. To support her, Christa drove early in the morning, when the cattle drink, to Duurwater to count the animals with the other committee members. “Then, we approached the poor households and talked to them about the different rules and encouraged them to stand up and talk in the meeting. In the meeting we would support them.”^8^

Taking the four dynamics together allows us to formulate a hypothesis: communities will only apply proportional equality when the state actively supports the poor and their interests. In all other cases, the three social dynamics described above favor numerical equality. As the correlation between the two variables, state interventions, and existence of proportional equality reveals (phi = −0.478, P = 0.000), the involvement of the state can explain the institutional outcome to a significant degree. In contrast, in communities where the state is only weakly involved the first three dynamics analyzed above are dominant and numerical equality prevails.

### Levels of success

We have outlined when and why different equality regimes evolve. Since the groundwater in northwestern Namibia comes from aquifers fed in Angola, ecological success cannot be measured locally. We, therefore, base our analysis of the success of the water management regimes on social indicators developed in cooperation with communities. These include (1) satisfaction with the rules, (2) satisfaction with the work of the water point committee (committee), (3) general level of satisfaction expressed concerning cooperation in the community, and (4) the general satisfaction with the water management in the community.

Table 1 shows how these variables and the sharing rules correlate. In general, the analysis reveals clearly that proportional equality (coded as ‘1’ in the dichotomous variable) leads to higher levels of satisfaction and success. All correlations are positive and significant. While the relationship is highest with the rules themselves, it holds true for the satisfaction with the committee work, and the cooperation in the community in an attenuated form as well. Above all, the general level of satisfaction with the water management in the community correlates positively with proportional equality. While this could have to do with higher reliability and better “performance” of boreholes that are managed under proportional equality, there is no correlation between the sharing rule and the susceptibility of the technological infrastructure and thus water access or supply (phi = −0.01, non-significant). Combined with our ethnographic observations, this indicates that success is mostly judged socially.

**Table 1:** Correlation between different indicators of perceived success and sharing agreements. The level of success was coded as follows: ‘1’ (unhappy or very unhappy), ‘2’ (it works okay), and ‘3’ (happy or very happy). Due to missing data, in all four correlations the N is lower than the total number of communities captured (60)

	Level of success											
	Institutional rules			Committee work			Cooperation in the community			Generally satisfied		
	1	2	3	1	1	1	1	2	3	1	2	3
Numerical	9	0	5	10	10	10	9	0	5	10	1	10
Proportional	1	2	18	3	3	3	1	2	18	3	3	24
Rank biserial correlation (rbc)	rbc = 0.559, P = 0.000			rbc = 0.427, P = 0.017			rbc = 0.406, P = 0.006			rbc = 0.389, P = 0.003		

### Economic consequences

As we have indicated, livestock is unequally distributed. Thus, with numerical equality in a hierarchical setting, where all individuals pay the same, the institutional regime must have economic consequences that diverge from equality. To explore these in depth, we developed a simulation model. The model starts with the distribution of livestock that we found among 80 households for which we did detailed livestock counts. Small and large stock are aggregated into one measure, livestock units (LSU), for which a head of cattle equals one LSU and small stock (goat, sheep) one sixth (Schnegg et al. 2013). For each month, we assume that herds grow by 2 % and that under numerical equality a household has to pay a constant water fee (200 N$). In order to pay, households sell animals at 4000 N$ per LSU. These numbers reflect local conditions. To explore how numerical equality affects different groups, we distinguish between three economic positions: the poor (< 20 LSU), those moderately well-off (>20 LSU < 100 LSU), and the rich (>100 LSU). The classification is based on wealth-ranking interviews and emic understanding of economic stratification. Among the 80 households, 21 were poor, 35 were moderately well-off, and 24 were rich.

**Fig. 1:**
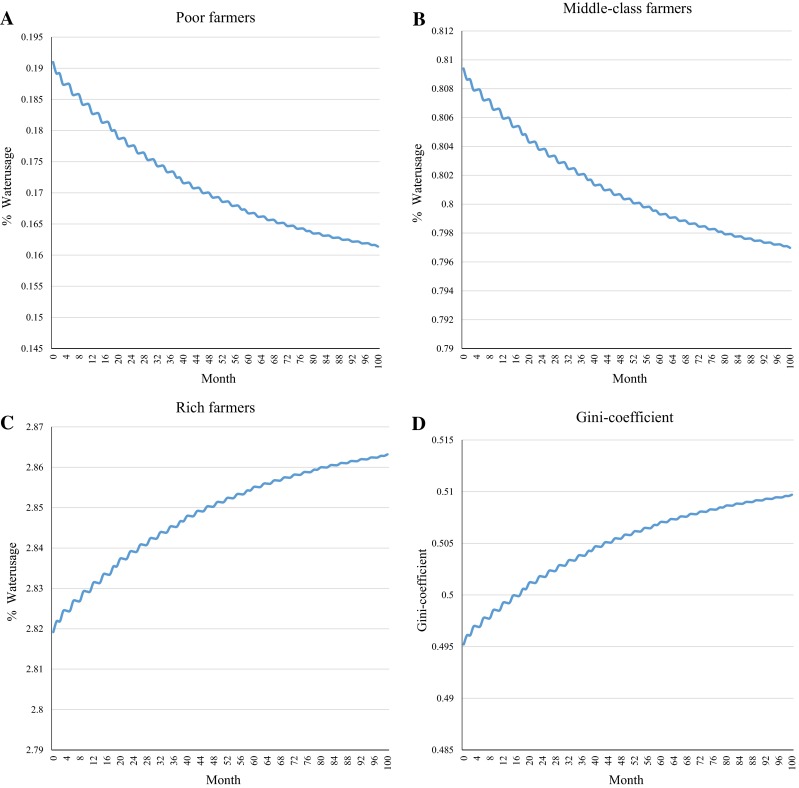
Simulation results. **a** Percentage of water used by an average poor household under a numerical equality rule over a period of 100 months. The percentage of the overall water consumption is very low and decreases. **b** Percentage of water used by an average middle-class household under a numerical equality rule over a period of 100 months. The percentage of the overall water consumption is low and decreases. **c** Percentage of water used by an average rich household under a numerical equality rule over a period of 100 months. The percentage of the overall water consumption is high and increases. **d** Gini coefficient applied to the distribution of livestock among all households. The Gini coefficient rises considerably during the 100-month period

Figure 1 shows the simulation results for the different economic groups over a period of 100 months. Figure 1a–c reports the proportion of water used by an average household in any of the three groups. While all households pay an equal amount to use the water, the amount used varies significantly between groups, and also changes with time. As the results reveal, at the beginning the share of water used by a rich household was already almost 15 times that used by its poor neighbor. That means that they get 15 times more water for the same contribution made. Over time, the amount of water used by the wealthy increases further, so after 100 months it is 18 times what the poor consumed. Since all households paid the same, the water consumption of the rich was subsidized by the other two groups—most significantly by those who owned the least.

Figure 1d investigates the wealth effects of these dynamics and shows the resulting Gini coefficient for livestock ownership for the population of 80 households. As the results reveal, the coefficient rises, indicating, *ceteris paribus*, that inequality increases. Thus, the poor and those in between not only subsidize the day-to-day water consumption of the richer part of the population, but also sponsor their future wealth.

## Discussion and conclusion

Even though the saliency of sharing rules has been recognized for some time, we know comparably little about what leads to the implementation of specific rules and what effects they have. Namibia currently offers the opportunity to investigate both relationships on a relatively large scale (Bollig and Menestrey Schwieger 2014; Schnegg and Linke 2015; Schnegg and Bollig 2016). To compare cost- and benefit-sharing arrangements, we introduce the distinction between numerical and proportional equality. Previously, research has assumed that in cases where benefits are unequally distributed, proportional equality is likely to prevail simply because it is generally perceived to be fair and would be beneficial for resource management (Ostrom 1990). Perhaps surprisingly, our results show that this is not always the case.

Proportional equality is considered fair by the largest part of the population. Equally, the correlation between different indicators of satisfaction and the rule type indicates that communities with proportional equality are more satisfied that is more successful. Hence, in terms of success, we confirm previous research that identified proportional equality to be more beneficial to the community as a whole (Ostrom 1990; Cox et al. 2010). At the same time, it often does not prevail. To explain why, we need to take the micro-politics of water governance into account. Those involve three salient intra-community dynamics (administration cost; wealth and bargaining power; multiplexity of ties and general norms of sharing) which all push the institutional regime toward numerical equality. These findings support recent work showing that natural resource management is embedded in past, present, and future social relationships (Cleaver 2012; De Koning and Cleaver 2012; Hall et al. 2014; Cleaver and de Koning 2015; Schnegg and Linke 2015).

At the same time, the state plays a decisive role. However, its role is not restricted to transferring global models of resource governance to national legislations. Through its bureaucrats and contracted NGOs, it remains an active agent in daily negotiations at the local level. Where the state remains actively involved, proportional equality is significantly more likely to be applied and to prevail.

The two equality rules have different effects. With proportional equality, everyone pays for their water usage by amount. Thus, the rule will not change the distribution of wealth in the community. By contrast, and as simulations reveal, numerical equality has far-reaching economic and social consequences. Both the poor and the moderately well-off subsidize the current water consumption of the rich. In the long run, they also assist in building up the future wealth of the latter.

These findings have political implications. Over recent decades, nation states have increasingly withdrawn from local resource management and self-governance (CBNRM) has become a guiding principle of many development efforts—the idea that turning ownership and responsibility over to local communities will empower them and help eradicate poverty (Ribot 1999; Blaikie 2006; Ribot et al. 2006). Left alone, we find that the rich typically succeed in establishing numerical equality, much to their own economic advantage (for a comparable observation see also Kumar 2002, p. 777). We demonstrate that these policies can have unintended economic consequences, especially in widening the gap between rich and poor. Only if the state remains in an active role can it ensure economic sustainability and help eradicate poverty.

## Footnotes

1 At the organizational level, two bodies are established. The Water Point Association (WPA) that usually includes all adult individuals living in the place. The WPA appoints a governance board, the Water Point Committee (WPC), which consists of five to seven members and is responsible for managing the daily concerns (Namibia 2004).

2 In some parts of southern Kunene, infrastructural problems are more common than in the northern part and are mainly caused by elephant herds; communities are supported by NGOs and state authorities to prevent destruction and undertake the necessary repairs.

3 Small and large stock are combined into one measure, livestock units (LSU), whereas a head of cattle equals one LSU and small stock (goat, sheep) one sixth.

4 The following analysis treats the six cases with an attenuated proportional rule as cases of a proportional rule.

5 While the rules differ, the total amount of money collected does not differ significantly with the institutional regime.

6 Interview conducted by M. Schnegg, 20.3.2014 (Fransfontein area).

7 Name is a synonym.

8 Interview conducted by M. Schnegg, 25.3.2014 (Fransfontein area).
